# Chronic spontaneous urticaria following ChAdOx1-S COVID-19 vaccination

**DOI:** 10.1007/s40629-022-00204-x

**Published:** 2022-03-07

**Authors:** Dan Suan, Adrian Y. S. Lee

**Affiliations:** 1 Department of Clinical Immunology and Allergy, Westmead Hospital, Hawkesbury Road, 2145 Westmead, NSW Australia; 2 Westmead Clinical School, University of Sydney, Westmead, NSW Australia; 3 Garvan Institute for Medical Research, Darlinghurst, NSW Australia; 4 Immunopathology, ICPMR and NSW Health Pathology, Westmead, NSW Australia

**Keywords:** Allergy, Coronavirus disease 2019, Adverse reactions, Prednisolone, Vaccine

A 39-year-old Filipino man presented 2 weeks following his second ChAdOx1‑S (Oxford/AstraZeneca) coronavirus disease 2019 (COVID-19) vaccination with complaints of widespread urticaria and swelling of his hands. These were spontaneous and occurred daily without specific physical or psychological triggers. His first ChAdOx1‑S vaccine was administered uneventfully one month prior. The patient had no history of atopic disorders and had never had urticaria.

**Fig. 1:**
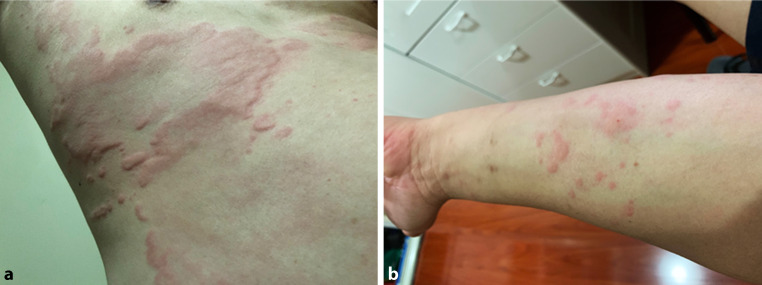
Coalescing urticarial lesions on the patient’s thorax (**a**) and forearm (**b**)*. *Informed, written consent has been obtained from the patient for the publication of his pictures

He had typical, raised urticarial lesions concentrated on his palms, upper limbs, abdomen, chest and sparing his face (Fig. 1). There was no dermatographism, appreciable angioedema of his face or body, and lung auscultation did not reveal any wheeze. There was neither lymphadenopathy nor hepatosplenomegaly.

Blood tests were unremarkable with a normal haematological profile without eosinophilia, normal biochemistry and a normal tryptase at 5.0 μg/L (< 11.5 μg/L). His thyroid studies, iron studies, autoimmune serology and serum electrophoresis/immunofixation were unremarkable. Rheumatoid factor was undetectable and his C3/C4 complements were mildly elevated.

He was commenced on a course of oral prednisolone (0.5 mg/kg) for 1 week and high-dose H1 antihistamines (cetirizine 20 mg twice daily) with some resolution of his symptoms. One week on, he reported the development of new urticarial lesions and was commenced on another course of prednisolone. He had a further relapse 3 weeks later (6 weeks post onset) and H2 antihistamines (nizatidine 150 mg twice daily) were added with good effect. He was initiated on a slower taper of oral prednisolone. The severity of the lesions continued to decline. At the 6‑month mark, he reported scattered daily urticarial lesions, but was able to control this with regular cetirizine tablets daily.

Allergy testing was limited to specific IgEs since his dependence on antihistamines and prednisolone precluded skin testing. He demonstrated negative (< 0.30 kU/L) tests to a range of grass and tree pollen, and chicken meat (a patient attribution). He was sensitised to house dust mites (3.66 kU/L) but there were no environmental variations to his urticaria.

Chronic spontaneous urticaria (CSU) is a disorder caused by the abnormal activation of mast cells and basophils by various triggers [1]. It is possible that the ChAdOx1‑S vaccination stimulated immune complexes and/or autoantibodies that led to emergence of our patient’s CSU, explaining the delay between vaccination and his symptoms. Supporting this theory, in patients with pre-existing CSU, cases of exacerbations following COVID-19 vaccination have been reported [2], suggesting the vaccine may cause perturbation of the immune system in a manner that promotes the pathogenesis of urticaria. Given CSU is a fairly common disease, an alternate hypothesis is that the vaccination and CSU occurred in this order purely by chance.

Cutaneous manifestations following COVID-19 vaccination have been well described in the literature, including both acute and chronic urticaria [3, 4]. CSU has been rarely reported. In one case, daily urticaria emerged 2 months following Comirnaty (Pfizer/BioNTech) COVID-19 vaccination administration but was highly responsive to cetirizine [4]. Another case detailed CSU shortly after ChAdOx1‑S vaccine, responsive to antihistamines and prednisolone, and achieved remission in 3 months [5]. In contrast, our patient still has not achieved remission 6 months after urticaria emergence and required multiple rounds of prednisolone.

Our patient has declined a booster vaccine for fear of exacerbation of his CSU. Although large studies have revealed rare recurrent cutaneous reactions on subsequent COVID-19 vaccines [6], there is little known about alternate vaccines to ones that have stimulated CSU. In one report [5], the patient had a mild flare of his ChAdOx1‑S vaccine-induced CSU in response to the Pfizer/BioNTech vaccine. Indeed, future reports and experience of vaccine-induced CSU should shed light on the natural history and safety of alternate vaccines.
